# Transition paths in single-molecule force spectroscopy

**DOI:** 10.1063/1.5004767

**Published:** 2017-12-21

**Authors:** Pilar Cossio, Gerhard Hummer, Attila Szabo

**Affiliations:** 1 Biophysics of Tropical Diseases Max Planck Tandem Group, University of Antioquia, Medellín, Colombia; 2 Department of Theoretical Biophysics, Max Planck Institute of Biophysics, 60438 Frankfurt am Main, Germany; 3 Institute of Biophysics, Goethe University Frankfurt, 60438 Frankfurt am Main, Germany; 4 Laboratory of Chemical Physics, National Institute of Diabetes and Digestive and Kidney Diseases, National Institutes of Health, Bethesda, Maryland 20892-0520, USA

## Abstract

In a typical single-molecule force spectroscopy experiment, the ends of the molecule of interest are connected by long polymer linkers to a pair of mesoscopic beads trapped in the focus of two laser beams. At constant force load, the total extension, i.e., the end-to-end distance of the molecule plus linkers, is measured as a function of time. In the simplest systems, the measured extension fluctuates about two values characteristic of folded and unfolded states, with occasional transitions between them. We have recently shown that molecular (un)folding rates can be recovered from such trajectories, with a small linker correction, as long as the characteristic time of the bead fluctuations is shorter than the residence time in the unfolded (folded) state. Here, we show that accurate measurements of the molecular transition path times require an even faster apparatus response. Transition paths, the trajectory segments in which the molecule (un)folds, are properly resolved only if the beads fluctuate more rapidly than the end-to-end distance of the molecule. Therefore, over a wide regime, the measured rates may be meaningful but not the transition path times. Analytic expressions for the measured mean transition path times are obtained for systems diffusing anisotropically on a two-dimensional free energy surface. The transition path times depend on the properties both of the molecule and of the pulling device.

## I. INTRODUCTION

Recently, we developed a quantitative theory of force spectroscopy experiments that accounts for the effects of the mesoscopic pulling device on the apparent rates of conformational transitions.^1^ Here, we adapt this theoretical framework to examine the effects of the measurement apparatus on the apparent transition path times. Transition paths are those segments of a trajectory where conformational transitions actually happen. The mean transition path time for protein folding was first determined experimentally by Chung and Eaton^2,3^ using single-molecule Förster resonance energy transfer (FRET) measurements. Then, using single-molecule force spectroscopy, Woodside and co-workers^4–7^ determined not only the mean but also the distribution of the transition path times. Motivated by the success of these experiments, we concentrate on the effect of the measurement device on the transition paths.

**FIG. 1.**
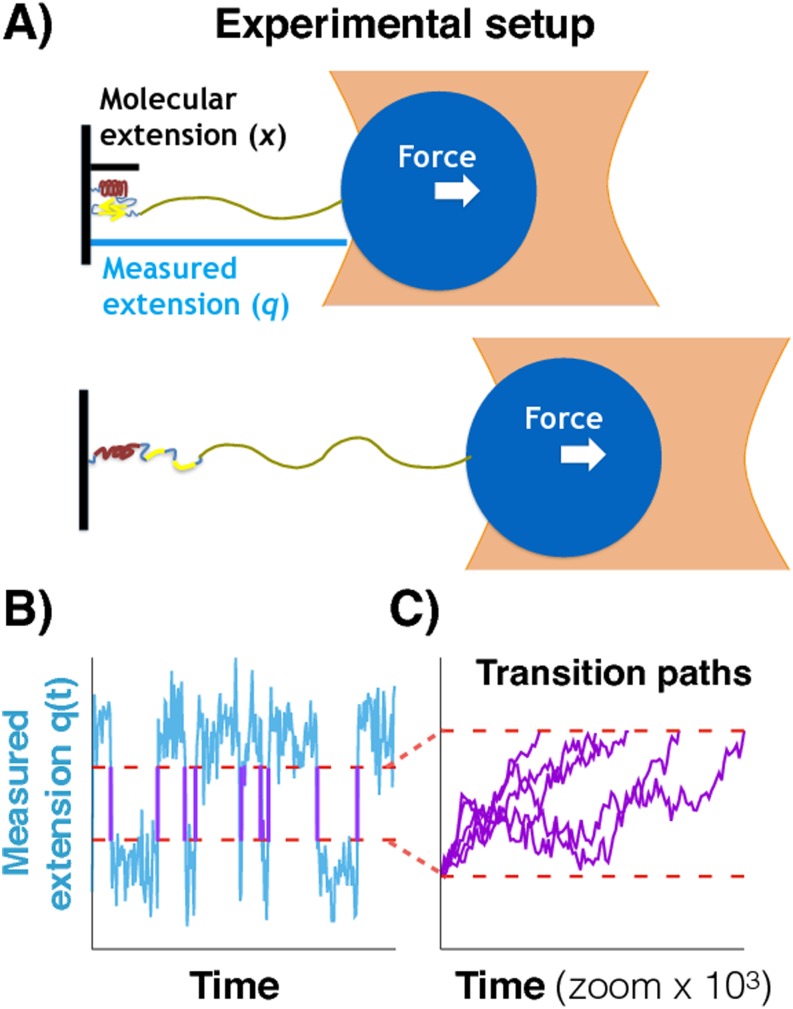
Transition paths from force spectroscopy experiments. (a) Representation of an optical tweezer experiment at constant force when the populations of the folded and unfolded states are equal. A molecular construct is attached via a typically long and soft polymeric linker to a mesoscopic pulling device. *x* is the end-to-end distance of the molecule, and *q* is the total measured extension. The fluctuations of the folded or unfolded molecule can be faster than those of the bead. (b) Measured extension *q* as a function of time *t* for the constant force experiments. The extension fluctuates about values characteristic of the folded and unfolded states with fast transitions between these conformational states. The transition paths (purple solid lines) pass directly between pre-defined limits (red lines). (c) Zoom-in on the transition paths aligned to start at the same time.

A schematic representation of a force spectroscopic experiment using a laser tweezer is shown in Fig. 1(a). A molecule (left) is attached via a soft polymer linker to a bead trapped in the focus of a laser beam. In the presence of a constant force, the molecule fluctuates between folded and unfolded conformations. The total measured extension *q*, of molecule plus linker, is monitored and plotted as a function of time in Fig. 1(b). In the folded (unfolded) state, the extension fluctuates about a small (large) value. Occasionally, there are conformational transitions, and the extension changes rapidly, as compared to the time spent in a state, from one reference value to the other. These transition paths are shown in purple in Fig. 1(b). Figure 1(c) zooms in on the transition paths, which have been aligned to start at the same time. Because of microscopic reversibility, the transition path ensembles for folding and unfolding are the same (i.e., if the direction of time is reversed, unfolding transitions in the trajectory become folding transitions and vice versa). For typical systems, transition path times are on the microsecond time scale, whereas the residence times (i.e., the time spent in a state before a transition occurs) are on the millisecond or slower time scale.

In this work, we study how the properties of the observed transition paths depend on the relative time scales of the fluctuations of molecular (*x*) and measured (*q*) extensions [Fig. 1(a)]. We begin in Sec. II A by summarizing the properties of the transition paths in one dimension (1D). Then, in Sec. II B, we consider the transition paths in two dimensions (2D) within the framework of anisotropic diffusion on a free energy surface that depends on both the molecular and measured extensions. For the regime in which the diffusion coefficient *D*_*x*_ of the molecular extension *x* is larger than the diffusion coefficient *D*_*q*_ of the measured extension *q*, we derive analytical expressions for the mean transition path time determined from the trajectories of the measured extension. We validate these expressions using Brownian dynamics simulations and find that the mean transition path time is inversely proportional to the “apparatus” diffusion coefficient *D*_*q*_ even in the regime where the measured transition rate is similar to the molecular rate. Finally, we discuss the implications of these results for the analysis of the transition paths obtained using single-molecule force spectroscopy.

## II. THEORY

### A. Transition paths for 1D diffusion

First, we summarize some results on the properties of transition paths for 1D diffusive dynamics. A transition path from *x* = *a* to *x* = *b* is defined as a segment of a trajectory *x*(*t*) that starts from *a* and reaches *b* directly, without first returning to *a*. The average duration of such a path in the presence of a potential *G*(*x*) is^8^

$$\begin{array}{ccc} \left\langle t_{\text{TP}}\left(a↔b\right)\right\rangle & = & \int_{a}^{b}dx\;e^{-\beta G\left(x\right)}\phi \left(x\right)\left(1-\phi \left(x\right)\right)dx\\ & & \times \;\int_{a}^{b}dx^{\prime }\;e^{\beta G\left(x^{\prime }\right)}/D\left(x^{\prime }\right), \end{array}$$ (1)

where *D*(*x*) is the position-dependent diffusion coefficient, *β* = 1/*k*_*B*_*T* is the reciprocal temperature, *k*_*B*_ is Boltzmann’s constant, and *ϕ*(*x*) is the committor,

$$\phi \left(x\right)=\frac{\int_{a}^{x}dy\;e^{\beta G\left(y\right)}/D\left(y\right)}{\int_{a}^{b}dy\;e^{\beta G\left(y\right)}/D\left(y\right)},$$ (2)

which is the probability of reaching *b* before *a*, starting from *x*.

For a harmonic barrier, *G*(*x*) = −*κx*^2^/2, and a constant diffusion coefficient, *D*(*x*) = *D*, the mean duration of a transition path between ±*L* obtained by evaluating the above integrals for large *L* is^9^

$$\left\langle t_{\text{TP}}\left(-L↔L\right)\right\rangle \approx \frac{\ln\left(e^{\gamma }\beta \kappa L^{2}\right)}{D\beta \kappa },$$ (3)

where *γ* ≈ 0.5772 is the Euler-Mascheroni constant. The free energy difference between *x* = ±*L* and the barrier top is $\Delta G^{‡}=\kappa L^{2}/2$. For fixed curvature *κ*, *L* can be eliminated in favor of $\Delta G^{‡}$, yielding the more familiar form of this equation. Alternatively, for fixed *L*, *κ* can be eliminated in favor of $\Delta G^{‡}$.

As an aside, we note that for barriers that fall off more steeply than harmonic, the average transition path times approach a finite value as *L*, or $\Delta G^{‡}$, go to infinity. For example, for the quartic barrier *G*(*x*) = −*κx*^4^, one obtains

$$\underset{L\to \infty }{\lim}\left\langle t_{\text{TP}}\left(-L↔L\right)\right\rangle =\frac{3{\left[\Gamma \left(5/4\right)\right]}^{2}}{2D\sqrt{2\beta \kappa }}\approx \frac{0.8714}{D\sqrt{\beta \kappa }},$$ (4)

where Γ(*x*) is the gamma function.

For a harmonic barrier, the distribution of transition path times for sufficiently high $\Delta G^{‡}$ can be approximated by^10^

$$p_{\text{TP}}\left(t\right)\approx \frac{\beta \kappa D\sqrt{\beta \Delta G^{‡}}}{erfc\left(\sqrt{\beta \Delta G^{‡}}\right)}\frac{e^{-\beta \Delta G^{‡}\coth\left(\beta \kappa Dt/2\right)}}{\sqrt{2\pi \sinh\left(\beta \kappa Dt\right)}\sinh\left(\beta \kappa Dt/2\right)},$$ (5)

where erfc(*x*) is the complementary error function. The above expression is exactly the distribution of conditional first passage times from *x* = −*L* to *x* = *L* with $\Delta G^{‡}=\kappa L^{2}/2$. Thus in this approximation, the system is allowed to recross the starting point (*x* = −*L*) before reaching *x* = *L*. Indeed, the corresponding mean time ($\int_{0}^{\infty }t\;p_{\text{TP}}\left(t\right)dt$) is always larger than the exact mean transition path time. However, as $\Delta G^{‡}$ or *L* → ∞, such recrossings become negligible, and in this limit, the mean time calculated using *p*_TP_(*t*) is exactly given by Eq. (3). For low barriers, say $\Delta G^{‡}$ = 1 *k*_*B*_*T*, the mean transition path time obtained using Eq. (5) is about twice larger than the exact value obtained from Eq. (1).

Recently, there has been considerable interest in the shape of transition paths.^11,12^ For a harmonic barrier, starting with the path integral representation of the propagator,^13^ it can be shown that the most probable path between ±*L* of duration *τ* is given by

$$\bar{x}\left(t|\tau \right)=\frac{L\sinh\left(\beta \kappa D\left(t-\tau /2\right)\right)}{\sinh\left(\beta \kappa D\tau /2\right)}$$ (6)

for 0 ≤ *t* ≤ *τ*. The most probable path of duration equal to the mean transition path time is $\bar{x}\left(t|\left\langle t_{\text{TP}}\left(-L↔L\right)\right\rangle \right)$. Using Eq. (3), this becomes

$$\bar{x}\left(t|\left\langle t_{\text{TP}}\left(-L↔L\right)\right\rangle \right)≃\frac{L\left(e^{\beta \kappa Dt}-\beta \kappa L^{2}e^{-\beta \kappa Dt+\gamma }\right)}{\beta \kappa L^{2}e^{\gamma }-1}$$ (7)

for large *L* (or $\Delta G^{‡}$).

**FIG. 2.**
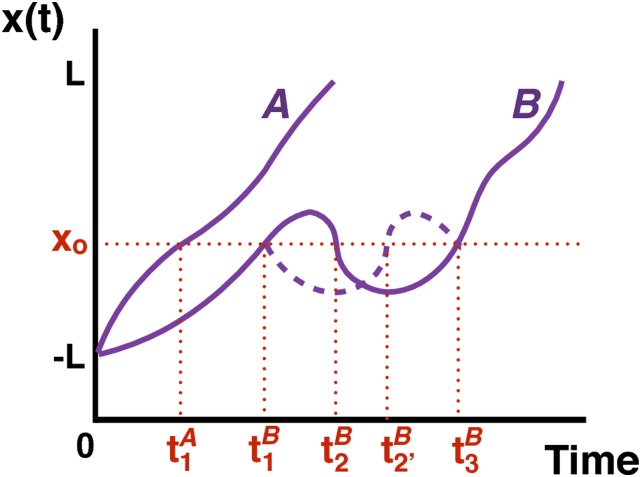
Example of two transition paths, *A* and *B*, aligned at −*L*. The first transition path (*A*) crosses *x*_*o*_ only once at time $t_{1}^{A}$; the second transition path (*B*) crosses *x*_*o*_ multiple times at $t_{1}^{B}$, $t_{2}^{B}$, and $t_{3}^{B}$. The dashed line shows the mirror-image path of transition path *B* between $t_{1}^{B}$ and $t_{3}^{B}$.

To test the accuracy of this approximation, one needs to average individual transition paths extracted, for example, from a long simulation. For the sake of simplicity, consider only two transition paths, *A* and *B*, that are aligned so that both are at *x* = −*L* at *t* = 0 as shown in Fig. 2. Because all paths have the same *x* range, but vary in their duration *t*, it is easier to calculate 〈*t*(*x*)〉, i.e., the average time that is assigned to position *x*. However, some care is required in defining this average. For the example shown in Fig. 2, one could calculate 〈*t*(*x*_*o*_)〉 by simply averaging all the time transition paths *A* and *B* cross *x*_*o*_, i.e., $\left(t_{1}^{A}+t_{1}^{B}+t_{2}^{B}+t_{3}^{B}\right)/4$. However, this procedure overestimates the contributions of transition path *B* which crosses *x*_*o*_ multiple times. The correct procedure is to first determine the average time $\bar{t}\left(x\right)$ for each trajectory and then average over all trajectories. For the example in Fig. 2, we have $\left\langle t\left(x_{o}\right)\right\rangle =\frac{1}{2}\left[{\bar{t}}^{A}\left(x_{o}\right)+{\bar{t}}^{B}\left(x_{o}\right)\right]=\frac{1}{2}\left[t_{1}^{A}+\frac{1}{3}\left(t_{1}^{B}+t_{2}^{B}+t_{3}^{B}\right)\right]$. For a path that crosses *x*_*o*_ at $t_{1}^{B},t_{2}^{B}$, and $t_{3}^{B}$, because of the microscopic reversibility and Markovian dynamics of *x*, there is a “mirror-image” path that crosses *x*_*o*_ at $t_{1}^{B},t_{2^{\prime }}^{B}$, and $t_{3}^{B}$ (dashed line in Fig. 2). The average value of *t*(*x*_*o*_) for these two paths is $\left(2t_{1}^{B}+2t_{3}^{B}+t_{2}^{B}+t_{2^{\prime }}^{B}\right)/6$. However, because of symmetry $t_{2}^{B}+t_{2^{\prime }}^{B}=t_{1}^{B}+t_{3}^{B}$ and so $\left\langle t\left(x_{o}\right)\right\rangle =\left(t_{1}^{B}+t_{3}^{B}\right)/2$, and it is sufficient to simply average the first and last crossing times. Thus, because of microscopic time reversibility, for trajectories that cross *x*_*o*_ for the first time at *t*_*f*_ and for the last time at *t*_*l*_, 〈*t*(*x*_*o*_)〉 = 〈*t*_*f*_ + *t*_*l*_〉/2 independent of the number of crossings in between. Therefore, to derive a simple analytic expression for 〈*t*(*x*)〉, note that on average the first time (*t*_*f*_) a transition path crosses *x* starting at −*L* is 〈*t*_*f*_〉 = 〈*t*_TP_(−*L* ↔ *x*)〉 since this segment of the trajectory is itself a transition path. Similarly 〈*t*_*l*_〉 = 〈*t*_TP_(−*L* ↔ *L*)〉 − 〈*t*_TP_(*x* ↔ *L*)〉, and thus

$$\left\langle t\left(x\right)\right\rangle =\frac{1}{2}\left(\left\langle t_{\text{TP}}\left(-L↔L\right)\right\rangle +\left\langle t_{\text{TP}}\left(-L↔x\right)\right\rangle -\left\langle t_{\text{TP}}\left(x↔L\right)\right\rangle \right)$$ (8)

for −*L* ≤ *x* ≤ *L*. As to be expected, 〈*t*(−*L*)〉 = 0 and 〈*t*(*L*)〉 = 〈*t*_TP_(−*L* → *L*)〉. Equation (8) is equivalent to that proposed by Makarov^12^ as a symmetrized version of the analytical expression found by Kim and Netz.^11^

It might also be interesting to consider the velocity along a transition path.^12^ For a transition path of duration *τ*, the most probable velocity can be obtained from Eq. (6),

$$\frac{d\bar{x}\left(t|\tau \right)}{dt}=\frac{L\beta \kappa D\cosh\left(\beta \kappa D\left(t-\tau /2\right)\right)}{\sinh\left(\beta \kappa D\tau /2\right)}.$$ (9)

Substitution of *τ* = 〈*t*_TP_(−*L* ↔ *L*)〉 in Eq. (1) gives the average “velocity” of the most probable path.

**FIG. 3.**
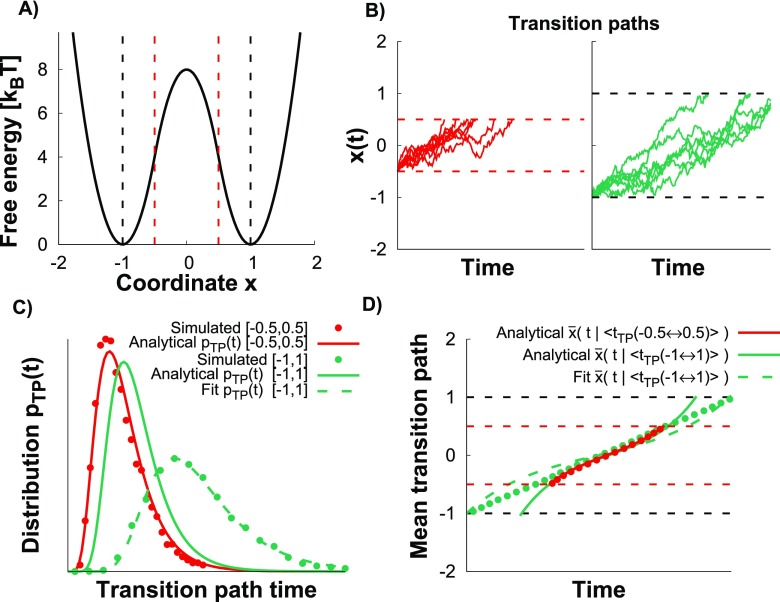
Transition paths over a 1D barrier. (a) 1D matched-harmonic potential surface with activation barrier 8 *k*_*B*_*T*. The transition paths are defined between *x* = −0.5 and 0.5 (*L* = 0.5) where the barrier is harmonic (vertical dashed red lines) and between the minima *x* = −1 and 1 (*L* = 1) where the barrier is anharmonic (vertical dashed black lines). Δ$G^{‡}$ that enters the various expressions is 4 *k*_*B*_*T* for *L* = 0.5 and 8 *k*_*B*_*T* for *L* = 1. (b) Transition paths from the Brownian dynamics simulations on the matched-harmonic potential (see Sec. III) for *L* = 0.5 (red) and *L* = 1 (green). The time scales are the same. (c) Distribution of transition path times for *L* = 0.5 and *L* = 1. The prediction from Eq. (5) with the exact *κ* and *D* is shown as the solid red line for *L* = 0.5 with Δ$G^{‡}$ = 4 *k*_*B*_*T* and as the solid green line for *L* = 1 with Δ$G^{‡}$ = 8 *k*_*B*_*T*. The fit of Eq. (5) is shown as the dashed green line for *L* = 1 by optimizing *D* using the exact *κ* and Δ$G^{‡}$ from the simulations. (d) The mean transition path shapes (see Sec. III) for *L* = 0.5 and *L* = 1 are compared to the analytic prediction Eq. (7) for *L* = 0.5 (solid red line) and *L* = 1 (solid green line). A fit of Eq. (7) with optimized *D* and exact *κ* is shown for *L* = 1 by the dashed green line.

To test the above expressions for harmonic and anharmonic barriers, we performed Brownian dynamics simulations on the matched-harmonic double-well potential shown in Fig. 3(a) (see Sec. III) with two sets of boundaries for the transition paths. The first set delimits the region where the barrier is exactly harmonic, between −0.5 and 0.5, i.e., *L* = 0.5; in the second set, between −1 and 1 with *L* = 1, the barrier also contains anharmonic segments [red and black dashed lines, respectively, in Fig. 3(a)]. The activation barrier $\Delta G^{‡}$ that enters the above formulas is 4 *k*_*B*_*T* for *L* = 0.5 and $\Delta G^{‡}$ = 8 *k*_*B*_*T* for *L* = 1. The examples of transition paths are shown in Fig. 3(b) for both sets. In Fig. 3(c), the distribution of transition path times for *L* = 0.5 is compared with the prediction of Eq. (5) evaluated for the same parameters used in the simulations (red points and line, respectively). The good agreement implies that $\Delta G^{‡}$ = 4 *k*_*B*_*T* is large enough for the high barrier approximation to apply. For *L* = 1, the prediction obtained from Eq. (5) with $\Delta G^{‡}$ = 8 *k*_*B*_*T* and the exact *κ* and *D* is inaccurate [solid green line Fig. 3(c)], as to be expected, since the transition path region is not harmonic. However, by optimizing *D*, with *κ* and $\Delta G^{‡}$ fixed to the exact values, a good fit can be obtained (dashed green line). The extracted *D* from the fit of *p*_TP_(*t*) is about 45% smaller than the exact value.

In Fig. 3(d), the mean transition path shape for *L* = 0.5 obtained from the simulations (red points) is in excellent agreement with the prediction of Eq. (7), and the prediction of Eq. (8) is virtually indistinguishable. In Fig. 3(d), we also show the mean transition path shape obtained for *L* = 1, the prediction of Eq. (7) using the exact parameters (solid green line), and the fit of Eq. (7) by optimizing *D* with *κ* fixed to the exact value (dashed green line). The fit does not completely capture the data, and the extracted *D* is 34% smaller than the exact *D*. By allowing also *κ* to float, the fit improves (see the supplementary material, Fig. 1). However, the fitted *κ* and *D* values are 98% and 73% lower than the exact values, respectively. Thus, if the barrier is exactly harmonic, the analytical expressions match well the results of the Brownian dynamics simulations; however, if this is not the case (here for *L* = 1), care should be taken when extracting model parameters.

### B. Transition paths in two dimensions

**FIG. 4.**
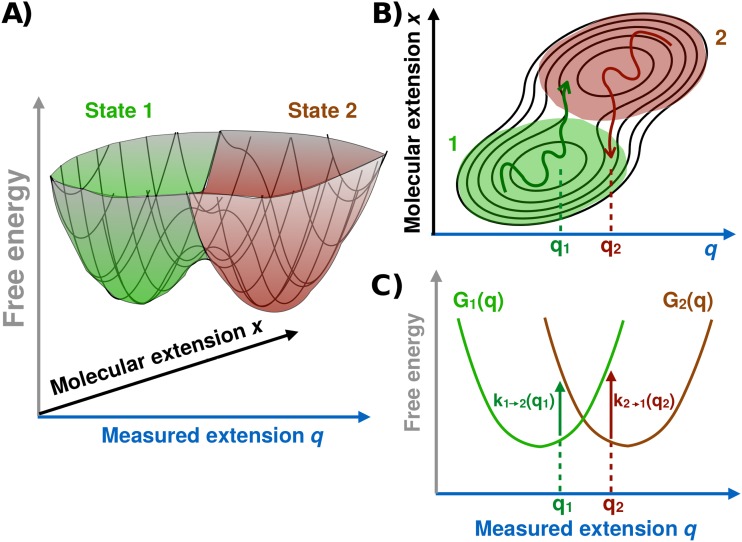
Surface hopping model. (a) Representation of the bistable 2D free energy surface as a function of the measured *q* and molecular *x* extensions. Green and brown surfaces represent conformational states 1 and 2, respectively. (b) Contour lines (black) of the free energy surface in the plane of *x* and *q*, together with two trajectory segments showing the surface hopping events at *q* ≈ *q*_1_ and *q*_2_, from state 1 (bottom, green) to state 2 (top, brown) and from state 2 to state 1, respectively. (c) Free energy profiles *G*_*i*_(*q*) obtained by integrating *x* over states *i* = 1 and 2. The dynamics on these 1D profiles is governed only by *D*_*q*_. The vertical arrows indicate the surface hopping transitions with rates *k*_1→2_(*q*) and *k*_2→1_(*q*), which are proportional to *D*_*x*_.

Consider a constant force experiment shown schematically in Fig. 1(a). The simplest description of this system that takes into account the pulling device is diffusion on a 2D free energy profile,^1,14–16^

$$G\left(x,q\right)=G_{o}\left(x\right)+\frac{\kappa_{l}}{2}{\left(x-q\right)}^{2}-Fq,$$ (10)

where *x* is the hidden molecular extension, *q* is the measured total extension [Fig. 4(a)], *κ*_*l*_ is the effective force constant of the linker, *G*_*o*_(*x*) is the bistable free energy surface of the molecule in the absence of force, and *F* is the applied force. *D*_*x*_ is the diffusion coefficient that describes the dynamics of *x* (which for the sake of simplicity is assumed to be the same for the unfolded and folded states). *D*_*q*_ is the diffusion coefficient along *q* which is essentially the diffusion coefficient of the mesoscopic bead (or the tip of a cantilever in an atomic force microscope) and therefore can be smaller than *D*_*x*_. The potential of mean force along *q*, *G*_A_(*q*), which can be obtained by binning the measured trajectory, is given by exp(−*βG*_A_(*q*)) ∝ ∫exp(−*βG*(*x*, *q*))*dx*.

Using the recent multidimensional reaction rate theory of Berezhkovskii *et al.*^17^ for large barriers and soft linkers, the rate constant for a conformational transition, *k*_MA_, is given by^1,15^

$$\frac{1}{k_{\text{MA}}}≃\frac{1}{k_{\text{L}}}+\frac{1}{k_{\text{A}}},$$ (11)

where *k*_L_ is the rate constant calculated using the Langer theory^18^ that depends on both *D*_*x*_ and *D*_*q*_, and *k*_A_ is the Kramers rate for diffusing on *G*_A_(*q*) with *D*_*q*_. This expression is valid for all *D*_*x*_ and *D*_*q*_. For fixed *D*_*x*_ as *D*_*q*_ decreases, the Langer rate reaches a plateau^19^ with $k_{\text{L}}\approx k_{\text{M}}\left(1-\kappa_{l}/|G_{o}^{\prime \prime }\left(x^{‡}\right)|\right)$, where $G_{o}^{\prime \prime }\left(x^{‡}\right)$ is the second derivative of the molecular free energy at the barrier top $x^{‡}$ and *k*_M_ is the molecular transition rate for 1D diffusion on the molecular free energy in the presence of force, $G_{M}\left(x\right)=G_{o}\left(x\right)\;-\;Fx$, with $D_{x}$. In this plateau region, when *D*_*q*_ is sufficiently large so that *k*_A_ ≫ *k*_M_, the measured rate constant *k*_MA_ is equal to the molecular rate constant *k*_M_ times a linker correction ($1-\kappa_{l}/|G_{o}^{\prime \prime }\left(x^{‡}\right)|$). When the linker is sufficiently soft so that $1\gg \kappa_{l}/|G_{o}^{\prime \prime }\left(x^{‡}\right)|$ then *k*_MA_ is essentially equal to *k*_M_. Otherwise the linker correction is significant. In other words, if the molecular barrier is sufficiently high, the measured rate *k*_MA_ is independent of *D*_*q*_ over a wide range of *D*_*q*_, and, in this regime, *k*_MA_ is equal to *k*_M_ aside from a small linker correction.

The dynamics simplifies when the pulling device relaxes slowly. When *D*_*q*_ is sufficiently small so that *k*_L_ becomes independent of *D*_*q*_, Berezhkovskii and Zitserman^20^ showed that, for high barriers, the diffusive dynamics on the 2D free energy surface can be described by a pair of coupled reaction diffusion equations (Fig. 4). The dynamics on these surfaces is determined only by *D*_*q*_, whereas the “hopping” rate constants depend only on *D*_*x*_. The relevance of this model for single-molecule force spectroscopy was pointed out in Ref. 14. In the regime where the hopping description is valid, the observed potential of mean force along the measured extension *q* contains very little information about the barrier region of the molecular potential surface along *x*.

Let *p*_1_(*q*, *t*) and *p*_2_(*q*, *t*) be the probabilities that the folded and unfolded states have total extension *q* at time *t*, respectively. These satisfy the coupled reaction diffusion equations,^14,20^

$$\frac{\partial p_{1}}{\partial t}=-k_{1\to 2}\left(q\right)p_{1}+k_{2\to 1}\left(q\right)p_{2}+D_{q}\frac{\partial }{\partial q}e^{-\beta G_{1}\left(q\right)}\frac{\partial }{\partial q}e^{\beta G_{1}\left(q\right)}p_{1},$$ (12a)

$$\frac{\partial p_{2}}{\partial t}=k_{1\to 2}\left(q\right)p_{1}-k_{2\to 1}\left(q\right)p_{2}+D_{q}\frac{\partial }{\partial q}e^{-\beta G_{2}\left(q\right)}\frac{\partial }{\partial q}e^{\beta G_{2}\left(q\right)}p_{2},$$ (12b)

where exp(−*βG*_*i*_(*q*)) ∝ $\int_{i}$ exp(−*βG*(*x*, *q*))*dx* with the integration being over the *i*th basin (*i* = 1, 2).

When the free energy, *G*(*x*, *q*), along *x* for fixed *q* has a double well shape, the hopping rates, *k*_1→2_(*q*) and *k*_2→1_(*q*), are calculated using Kramers theory for fixed *q*, e.g., $1/k_{1\to 2}\left(q\right)=\int_{1}dxe^{-\beta G\left(x,q\right)}\int_{‡}dx^{\prime }e^{\beta G\left(x^{\prime },q\right)}/D_{x}$, where “1” and “‡” indicate integration over the well of surface 1 and the barrier region, respectively. To evaluate these integrals for high barriers, we expand the 2D free energy to second order about the extrema and evaluate the resulting Gaussian integrals from *x* = −∞ to *x* = ∞. In this way, we find that $\exp\left(-\beta G_{i}\left(q\right)\right)\propto {\left(2\pi /\left(\beta \left(G_{o}^{\prime \prime }\left(x_{i}\right)+\kappa_{l}\right)\right)\right)}^{1/2}\exp\left(-\beta G_{M}\left(x_{i}\right)-\beta \kappa_{i}{\left(q-x_{i}-F/\kappa_{l}\right)}^{2}/2\right)$, where *x*_*i*_ is the location of the *i*th minimum (*i* = 1, 2) of *G*_*M*_(*x*), and $1/\kappa_{i}=1/G_{o}^{\prime \prime }\left(x_{i}\right)+1/\kappa_{l}$. The hopping rate is given by

$$\begin{array}{ccc} k_{1\to 2}\left(q\right) & = & \frac{\beta D_{x}}{2\pi }\sqrt{\left(\kappa_{l}+G_{o}^{\prime \prime }\left(x_{1}\right)\right)|\kappa_{l}+G_{o}^{\prime \prime }\left(x^{‡}\right)|}\\ & & \times \;e^{-\beta \left[\Delta G_{\text{M}}^{‡}+\frac{\kappa^{‡}}{2}{\left(q-x^{‡}-\frac{F}{\kappa_{l}}\right)}^{2}-\frac{\kappa_{1}}{2}{\left(q-x_{1}-\frac{F}{\kappa_{l}}\right)}^{2}\right]}, \end{array}$$ (13)

where $x^{‡}$ is the saddle point on *G*_*M*_(*x*) between *x*_1_ and *x*_2_, $\Delta G_{\text{M}}^{‡}$ is the molecular activation free energy in the presence of force for well 1, and $1/\kappa^{‡}=1/G_{o}^{\prime \prime }\left(x^{‡}\right)+1/\kappa_{l}$. *k*_2→1_(*q*) is given by flipping indices 1 and 2 in the above expression and replacing $\Delta G_{\text{M}}^{‡}$ by the molecular activation barrier in the presence of force for well 2. It can be shown that the rates *k*_*i*→*j*_(*q*) in Eq. (13) are independent of *F* and satisfy detailed balance with *k*_1→2_(*q*) exp(−*βG*_1_(*q*)) = *k*_2→1_(*q*) exp(−*βG*_2_(*q*)).

In general, to find the overall transition rates, Eqs. (12a) and (12b) must be solved numerically or equivalently, the corresponding stochastic equations must be simulated (see Sec. III). However, the problem simplifies when the hopping rates between surfaces (which are proportional to *D*_*x*_) are either much faster or much slower than the relaxation times on the two surfaces (which are inversely proportional to *D*_*q*_). When the hopping rates are so small that the system relaxes to local equilibrium before a jump occurs, the dynamics can be described by a two-state kinetic model with rates

$$\left\langle k_{i\to j}\right\rangle =\frac{\int k_{i\to j}\left(q\right)e^{-\beta G_{i}\left(q\right)}dq}{\int e^{-\beta G_{i}\left(q\right)}dq},$$ (14)

which depend only on *D*_*x*_. It has been shown^20^ that for high barriers and soft linkers, this rate is equal to the Langer rate in the limit that *D*_*q*_ → 0. In the opposite limit, when the hopping rates are much faster than the relaxation on the two free energy surfaces, the reaction diffusion equations, Eqs. (12a) and (12b), reduce to a 1D diffusion equation involving the potential of mean force *βG*_A_(*q*) = −ln(exp(−*βG*_1_(*q*)) + exp(−*βG*_2_(*q*))). Therefore, in the *D*_*q*_ → ∞ limit, the rates can be obtained using the Kramers theory for diffusion along *q* with diffusion coefficient *D*_*q*_ and potential of mean force *G*_A_(*q*).

We now turn to the calculation of the mean transition path time between *q* = *a* on surface 1 and *q* = *b* on surface 2 in these two limits. When the hopping rates are fast, we can simply use Eq. (3) with *G*(*x*) → *G*_A_(*q*) and *D* → *D*_*q*_. In the opposite limit, when Eq. (14) holds and the system is in the Langer plateau region (see Fig. 3 in Ref. 1), where the rate *k*_MA_ is essentially independent of *D*_*q*_, a transition path between *a* on surface 1 and *b* on surface 2 involves just a single hop (i.e., one can ignore repeated crossings). Let us assume that only a negligible number of transitions occur outside the interval [*a*, *b*]. Imagine that the system has jumped at *q* from surface 1 to surface 2 [e.g., Fig. 4(c) for *q* = *q*_1_]. When the hopping rates are so slow that the system will not jump back to surface 1 before reaching *q* = *b*, then the duration of this fragment of the trajectory is just the mean first passage time to reach *b* from *q* on surface 2, i.e., $\left\langle t_{\text{MFPT}}^{\left(2\right)}\left(q\to b\right)\right\rangle$, where the superscript indicates surface 2. Similarly if the system jumps from surface 2 to surface 1 and does not jump back before reaching *q* = *a*, then the mean duration of this segment is $\left\langle t_{\text{MFPT}}^{\left(1\right)}\left(q\to a\right)\right\rangle$. Now, due to microscopic reversibility, the duration of a transition path from *a* to *b* is the same as from *b* to *a* so that the average duration of a path from *q* = *a* on surface 1 to *q* = *b* on surface 2 that crosses between the two surfaces only once at *q* is $\left\langle t_{\text{MFPT}}^{\left(1\right)}\left(q\to a\right)\right\rangle +\left\langle t_{\text{MFPT}}^{\left(2\right)}\left(q\to b\right)\right\rangle$. Here we implicitly assumed that the paths between *a* and *b* remaining on the same surface are not considered to be the transition paths and, hence, not counted as such. When the hopping rates are very slow, the probability that a jump occurs at *q* in either direction is $p\left(q\right)=k_{1\to 2}\left(q\right)e^{-\beta G_{1}\left(q\right)}/\int_{a}^{b}k_{1\to 2}\left(q\right)e^{-\beta G_{1}\left(q\right)}dq=k_{2\to 1}\left(q\right)e^{-\beta G_{2}\left(q\right)}/\int_{a}^{b}k_{2\to 1}\left(q\right)e^{-\beta G_{2}\left(q\right)}dq$. Thus, in the regime that *k*_MA_ is independent of *D*_*q*_, the mean transition path time measured along *q* is

$$\begin{array}{ccc} {\left\langle t_{\text{TP}}\left(a↔b\right)\right\rangle }_{\text{MA}} & \approx & \int_{a}^{b}p\left(q\right)\left[\left\langle t_{\text{MFPT}}^{\left(1\right)}\left(q\to a\right)\right\rangle \right.\\ & & +\;\left.\left\langle t_{\text{MFPT}}^{\left(2\right)}\left(q\to b\right)\right\rangle \right]dq\\ & = & \frac{1}{D_{q}}\int_{a}^{b}p\left(q\right)\left[\int_{a}^{q}e^{\beta G_{1}\left(y\right)}dy\int_{y}^{\infty }e^{-\beta G_{1}\left(z\right)}dz\right.\\ & & +\;\left.\int_{q}^{b}e^{\beta G_{2}\left(y\right)}dy\int_{-\infty }^{y}e^{-\beta G_{2}\left(z\right)}dz\right]dq, \end{array}$$ (15)

which is inversely proportional to *D*_*q*_. This result means that for *D*_*q*_ < *D*_*x*_, there exists a regime of diffusion anisotropy where, aside from a small linker correction, the measured overall transition rate is the same as the molecular one (i.e., proportional to *D*_*x*_) but the transition paths along *q* are not the same as the molecular ones because they are determined by the diffusion coefficient *D*_*q*_ of the apparatus and not that of the molecule, *D*_*x*_. This point is consistent with our previous simulations (see the inset of Fig. 3 in Ref. 1). It is also supported by the recent work of Makarov,^21^ which showed that for a 2D surface with an entropic barrier, a 1D projection can give the correct transition rate but an incorrect transition path ensemble.

## III. METHODS

### A. 1D Brownian dynamics simulations

To validate the analytical expressions of Sec. II A, we performed Brownian dynamics simulations by numerically solving the 1D overdamped Langevin equation, *x*_*n*+1_ = *x*_*n*_ − Δ*tDβG′(x_*n*_)* + (2*D*Δ*t*)^1/2^*R*(*n*), where *D* is the diffusion coefficient, *G*′(*x*) is the derivative of the 1D free energy surface, Δ*t* is the time step, and *R*(*n*) is an uncorrelated Gaussian random number with zero mean and unit variance. The free energy surface is the bistable matched-harmonic potential with *βG*(*x*) = −16*x*^2^ + 8 for 0 ≤ |*x*| ≤ 1/2, and 16(|*x*| − 1)^2^ for 1/2 < |*x*|, which corresponds to a barrier height of 8 *k*_*B*_*T*. The time step was Δ*t* = 5 × 10^−6^/*D*.

### B. 2D Brownian dynamics simulations

We performed 2D Brownian dynamics simulations by numerically solving the 2D overdamped Langevin equation using the free energy surface given in Eq. (10), similarly as in Ref. 1. A constant force *F*_1/2_ is applied to make the populations of the folded and unfolded states equal. The molecular free energy surface is chosen to be the bistable matched-harmonic with $G_{o}\left(x\right)-F_{1/2}x=\Delta G_{M}^{‡}f\left(\right.x/\Delta x^{‡})$, where *f*(*x*) = −2*x*^2^ for 0 ≤ |*x*| ≤ 1/2 and *f*(*x*) = 2(|*x*| − 1)^2^ − 1 for 1/2 < |*x*|. $\Delta G_{M}^{‡}=8.1\;k_{B}T$ is the activation barrier of the molecule in the presence of a force *F*_1/2_, and Δ$x^{‡}$ = 3/2 is the distance to the transition state at *F*_1/2_. The molecule is coupled to the apparatus through a harmonic linker *κ*_*l*_ = 2.6 *k*_*B*_*T*/[*x*^2^] (where [*x*^2^] are units of distance squared), and the ratio of the linker and the molecular force-constants is $\kappa_{l}/|G_{o}^{\prime \prime }\left(x^{‡}\right)|\approx 1/6$. These parameters are chosen similar to those found for the 20TS06/T4 DNA hairpin.^16^ Simulations were also performed for the free energy surface used in Ref. 1, which has a larger activation barrier $\Delta G_{M}^{‡}=16\;k_{B}T$, and for $\kappa_{l}/|G_{o}^{\prime \prime }\left(x^{‡}\right)|=1/8$. The time step was Δ*t* = 5 × 10^−4^/*D*_*x*_. Approximately 7000 transition paths each were produced for a series of *D*_*x*_/*D*_*q*_ ratios.

### C. Hopping simulations

We simulated trajectories corresponding to coupled reaction diffusion equations, Eqs. (12a) and (12b), using a hybrid Brownian dynamics/Monte Carlo algorithm.^14^ The positions on each surface were determined by a 1D overdamped Langevin equation $q_{n+1}=q_{n}-\Delta tD_{q}\beta G_{i^{\prime }}\left(q_{n}\right)+{\left(2D_{q}\Delta t\right)}^{1/2}R\left(n\right)$, where *i* = 1, 2 indexes the potential surface, Δ*t* is the time step, and *R*(*n*) is an uncorrelated Gaussian random number with zero mean and unit variance. The trajectory stays on surface *i* at *q*_*n*+1_ with probability *p* = 1 − exp(−(*k*_*i*→*j*_(*q*_*n*_) + *k*_*i*→*j*_(*q*_*n*+1_))Δ*t*/2) and jumps to surface *j* at *q*_*n*+1_ with probability 1 − *p*. The time step was Δ*t* = 5 × 10^−4^/*D*_*x*_. The surface hopping rates, Eq. (13), were calculated for the parameters of the corresponding 2D Brownian dynamics model. Approximately 7000 transition paths each were produced for a series of *D*_*x*_/*D*_*q*_ ratios.

### D. Transition paths from simulations

The 1D Brownian dynamics simulations were analyzed by monitoring the *x* coordinate. The trajectories from the 2D Brownian dynamics and surface hopping simulations were analyzed by monitoring the measured extension *q* alone. Similar to experimental traces, the trajectories from the simulations were averaged over a short time window of 60Δ*t*. We found it particularly useful to smooth the traces from the 2D and hopping simulations that had a fast *D*_*q*_ (i.e., *D*_*q*_ ≫ *D*_*x*_) and showed large fluctuations about the mean (see the supplementary material, Fig. 2). For the 2D and hopping models, transition paths were defined as those parts of the smoothed trajectories that started from *q* = −*L* and crossed *q* = *L* before returning back to −*L*. The transition path time is the duration of each path, i.e., the time it takes to reach directly *L* starting from −*L*. The mean transition path time is the average duration of all paths. To calculate the mean transition path shape, we aligned the transition paths at the lower limit and discretized them along the extension. To avoid overweighting the contributions of each individual trajectory, due to recrossing at *x*_*o*_ (Fig. 2), we calculated for each transition path the mean time at *x*_*o*_, $\bar{t}\left(x_{o}\right)$. For example in Fig. 2, $\bar{t}\left(x_{o}\right)$ is $t_{1}^{A}$ for transition path *A* and $\left(t_{1}^{B}+t_{2}^{B}+t_{3}^{B}\right)/3$ for transition path *B*. The mean time assigned to *x*_*o*_ is $\left\langle t\left(x_{o}\right)\right\rangle =\sum_{j}{\bar{t}}_{j}\left(x_{o}\right)/N$, where the index *j* runs over the transition paths and *N* is the total number of transition paths.

## IV. RESULTS AND DISCUSSION

**FIG. 5.**
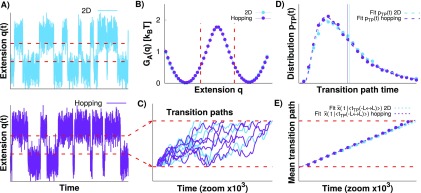
Comparison of the 2D Brownian dynamics and surface hopping models. (a) Typical *q*(*t*) trajectories from simulations with *D*_*x*_/*D*_*q*_ = 10 of 2D Brownian dynamics (blue) and of the surface hopping dynamics (purple), using free energy parameters that are similar to those of the DNA hairpin 20TS06/T4^16^ (see Sec. III for details). The molecular barrier height is 8.1 *k*_*B*_*T*. (b) Potential of mean force along the measured extension *q*, *G*_A_(*q*). The barrier height along *q* is 1.9 *k*_*B*_*T*. The limits of the transition paths are shown as dashed red lines. (c) Zoom-in on the transition paths with examples for the 2D (blue) and hopping (purple) transition paths aligned at the lower limit. (d) Distribution of the transition path times (symbols) together with fits of Eq. (5) and mean transition path time (vertical lines). (e) Mean transition path shape from the simulations with fits of Eq. (7) for the 2D Brownian dynamics (blue) and the surface hopping dynamics (purple).

We verified that the 2D and surface hopping simulation results (see Sec. III) are equivalent in the regime of slow apparatus diffusion, *D*_*x*_ > *D*_*q*_. In Fig. 5(a), we compare a trajectory from the 2D model (blue) to the one from the surface hopping model (purple). The ratio of the molecular and apparatus diffusion coefficients is *D*_*x*_/*D*_*q*_ = 10. The trajectories from the 2D and hopping simulations are qualitatively similar. In Fig. 5(b), we show the potential of mean force along *q*, *G*_A_(*q*), for both models. Notwithstanding that a Gaussian integral approximation has been used for *G*_*i*_(*q*) in the hopping model, the potentials of mean force are remarkably similar. The transition path limits are shown as the dashed red lines. In Fig. 5(c), we show the examples of transition paths from the simulations for the 2D and surface hopping models. Qualitatively the sets of transition paths seem indistinguishable, and their equivalence is confirmed by comparing the distribution of transition path times, shown in Fig. 5(d). Although Eq. (5) for *p*_*TP*_(*t*) is not expected to be valid here, since the system is not diffusing on a 1D profile, it is of interest to use this expression to fit the data by varying *κD* and $\Delta G^{‡}$. The fits are remarkably good [Fig. 5(d)], but the extracted parameters are different from those of the molecule and similar to those that describe transition paths on the potential of mean force along *q* [i.e., ${\left(\Delta G^{‡}\right)}_{fit}\approx 2\;k_{B}T$ and ${\left(\kappa D\right)}_{fit}\approx 0.02|G_{o}^{\prime \prime }\left(x^{‡}\right)|D_{x}\approx 1.06|G_{A}^{\prime \prime }\left(q^{‡}\right)|D_{q}$ where $\left|G_{A}^{\prime \prime }\left(q^{‡}\right)\right|$ is the barrier stiffness of *G*_*A*_(*q*)]. The barrier thus matches that of *G*_A_(*q*) in Fig. 5(b) and (*κD*)_*fit*_ matches $|G_{A}^{\prime \prime }\left(q^{‡}\right)|D_{q}$, i.e., the fits report primarily on the apparatus dynamics, not on the transition dynamics of the molecule. If we compare the mean transition path time for both models [vertical lines in Fig. 5(d)] to the molecular mean transition path time along *x*, we find that the mean transition path times for the 2D and hopping models are about an order of magnitude larger than that of the molecule.

In Fig. 5(e), we show the mean transition path shape for both simulations with fits of the analytical expression [Eq. (7)]. The mean shapes for the hopping and 2D models are practically indistinguishable, and the analytic expression fits the data well. However, the fits reveal that the extracted parameters *κ* and *D* are again similar to those of the apparatus.

**FIG. 6.**
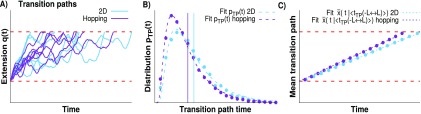
Results from the 2D model and surface hopping simulations when the diffusion coefficients of molecule and apparatus are equal, *D*_*x*_/*D*_*q*_ = 1. (a) Transition paths from the 2D (blue) and hopping (purple) simulations. The dashed red lines are the transition path limits. (b) Distribution of transition path times together with fits of Eq. (5) and mean transition path time (vertical lines). (c) The mean transition path shape with fits of Eq. (7) shown for both simulations.

In Fig. 6, we compare the results from the 2D and hopping simulations when the diffusion coefficient of the molecule is equal to that of the apparatus, *D*_*x*_/*D*_*q*_ = 1. Qualitatively, the transition paths from both models are similar [Fig. 6(a)]. However, the probability distribution of transition path times and its mean [Fig. 6(b)] show small differences. The mean transition path time for the hopping model is slightly faster than that for the 2D Brownian dynamics simulations. However, if we compare the mean transition path time for the 2D and hopping models to the mean molecular transition path time along *x*, we find that these are 3.7 and 3.2 times greater, respectively. The mean transition path shape, shown in Fig. 6(c), confirms that the transition paths for the hopping model are slightly faster than those for the 2D model. These results show that the surface hopping and 2D models exhibit essentially the same dynamics even when *D*_*x*_ = *D*_*q*_.

**FIG. 7.**
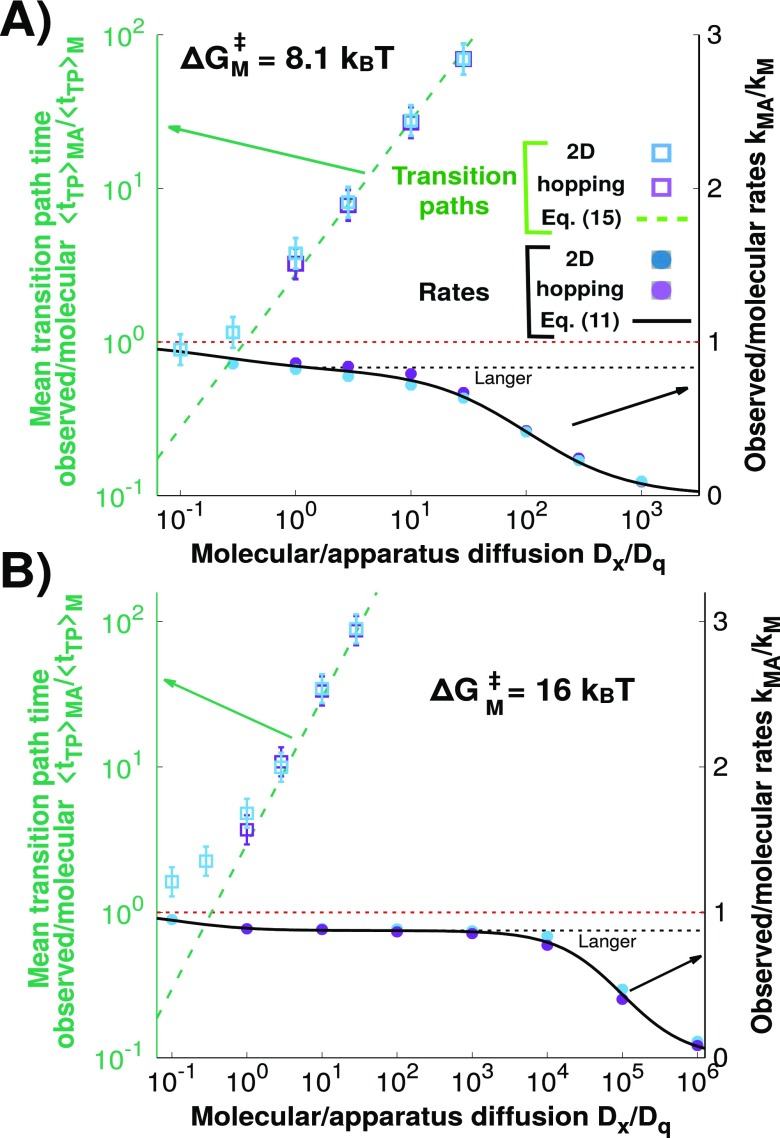
The mean observed transition path time depends on the apparatus diffusion coefficient. (Left axis; green; logarithmic scale) The open square symbols show the mean measured transition path time along *q*, ${\left\langle t_{\text{TP}}\right\rangle }_{\text{MA}}$, normalized by the mean molecular transition path time along *x*, ${\left\langle t_{\text{TP}}\right\rangle }_{\text{M}}$, as a function of the ratio of molecular and apparatus diffusion coefficients *D*_*x*_/*D*_*q*_ for the 2D (blue) and hopping (purple) simulations. The analytic prediction equation (15) is shown as a dashed green line. The horizontal dashed red line indicates that ${\left\langle t_{\text{TP}}\right\rangle }_{\text{MA}}$ and ${\left\langle t_{\text{TP}}\right\rangle }_{\text{M}}$ are equal. (Right axis; black; linear scale) Ratio of the measured and molecular transition rates, *k*_MA_/*k*_M_, for the 2D and hopping models, together with the prediction from Eq. (11) and the Langer theory (solid and dashed black lines, respectively). The transition rates and mean transition path times for the 2D and hopping simulations are similar for *D*_*x*_/*D*_*q*_ ≥ 1. In this regime, the prediction of Eq. (15) coincides with the results from the simulations. The transition path times and rates are shown (a) for the potential surface parameters chosen to be similar to those of the DNA hairpin 20TS06/T4^16^ and (b) for a higher barrier of 16 *k*_*B*_*T* and a softer linker $\kappa_{l}/|G_{o}^{\prime \prime }\left(x^{‡}\right)|=1/8$.

For slow apparatus dynamics, *D*_*q*_ < *D*_*x*_, the mean time of transition paths monitored along *q*, ${\left\langle t_{\text{TP}}\right\rangle }_{\text{MA}}$, depends strongly on the apparatus dynamics. We find that ${\left\langle t_{\text{TP}}\right\rangle }_{\text{MA}}$ is accurately given by Eq. (15) and grows as the reciprocal of the apparatus diffusion coefficient, 1/*D*_*q*_ (Fig. 7, left axis). The analytic expression [Eq. (15)] provides an excellent estimate of ${\left\langle t_{\text{TP}}\right\rangle }_{\text{MA}}$ both for the hopping model and for 2D diffusion for *D*_*q*_ < *D*_*x*_. We also show the ratio of the measured and molecular transition rates, *k*_MA_/*k*_M_, as a function of *D*_*x*_/*D*_*q*_ (Fig. 7, right axis). We find that the transition rates for both simulations are within computational error for *D*_*x*_/*D*_*q*_ ≥ 1 and the hopping model accurately captures the 2D model results. Therefore, we recover the Langer plateau described in Ref. 1, in which the measured rate *k*_MA_ does not significantly depend on *D*_*q*_. By contrast, in this regime, ${\left\langle t_{\text{TP}}\right\rangle }_{\text{MA}}$ is almost linearly dependent on $D_{q}^{-1}$ and independent of *D*_*x*_. These results do not change significantly for a higher molecular barrier of 16 *k*_*B*_*T* instead of 8.1 *k*_*B*_*T* [Fig. 7(b)].

If the diffusion coefficient of the apparatus becomes faster than that of the molecule, the transition path times obtained from observed *q* trajectories become quantitatively correct (Fig. 7). One may ask why for *D*_*q*_ > *D*_*x*_, we obtained estimates of the transition path time from the *q* trajectories of the 2D simulations that agree quite well with those from the hidden molecular transitions along *x*. This behavior is expected theoretically because, in this regime, the 2D diffusion equation is reduced to quasi-1D diffusion along x that involves the molecular potential of mean force. In practice, we need to average the *q* trajectories over multiple time frames, as is often done also with experimental trajectories. This averaging removes fast *q* dynamics (see the supplementary material, Fig. 2) and produces a smooth trajectory $\bar{q}=\bar{q}\left(x\left(t\right)\right)$ that is conditioned on the comparably slow *x*. In this fast *q* regime, one can thus resolve transition events by averaging away fast *q* fluctuations.

**FIG. 8.**
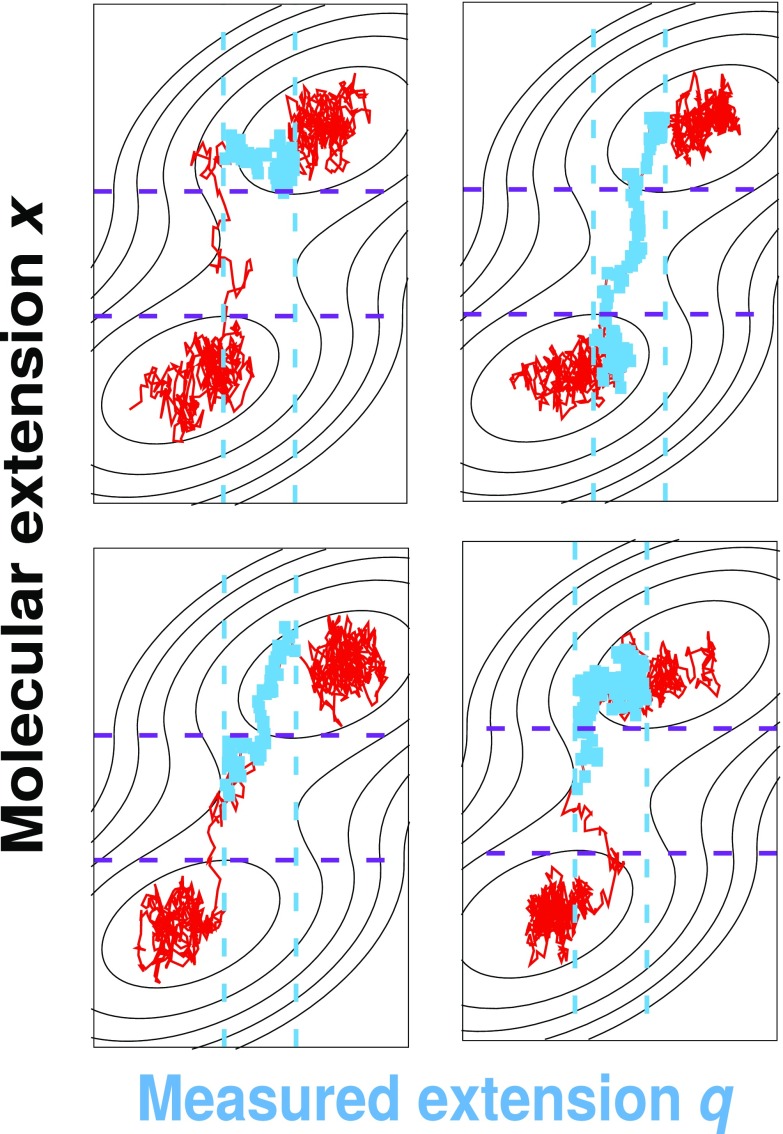
Examples of 2D transition paths (solid red lines) from the 2D Brownian dynamics simulations with *D*_*x*_/*D*_*q*_ = 1 as a function of the total measured extension *q* and the molecular extension *x*. The blue squares indicate the transition paths selected by analyzing the dynamics only along the measured extension *q*. Blue and purple dashed lines indicate the transition path limits along *q* and *x*, respectively. Note that in the top left panel, the *q* transition path (blue) has little to do with crossing the saddle but reflects the relaxation of the total extension after a molecular transition has taken place.

The fundamental reason for why it is difficult to obtain the transition path times and molecular free energy barriers for *D*_*q*_ < *D*_*x*_ is the same: the measured extension *q* is an imperfect estimator of the molecular extension *x*. This problem is illustrated in Fig. 8, which compares the true 2D transition paths from the 2D Brownian dynamics simulations for *D*_*x*_ = *D*_*q*_ (red lines) to the transition paths that were identified solely from the *q* dynamics (blue squares). As shown, the overlap of the true and estimated transition paths is poor because the boundaries along *q* (dashed vertical blue lines) invariably cut through the populated regions in both states. In other words, if one only knows *q*, it is difficult to decide when a transition path starts and when it ends.

It should be emphasized that the examples in Fig. 7 were chosen to reflect scenarios in which the influence of the apparatus is relatively small. For stiffer linkers and lower activation energies, the situation deteriorates because the Langer plateau (where the rate is essentially independent of *D*_*q*_) becomes narrower. For example, when $\Delta G_{M}^{‡}=4$ *k*_*B*_*T* (a relatively fast folder), the plateau essentially disappears,^17^ and when *D*_*x*_/*D*_*q*_ = 10, one cannot even extract an accurate molecular transition rate from the observed trajectories. In this case, both the measured transition paths and rates are well described by a model where the system diffuses on the measured potential of mean force [i.e., *G*_*A*_(*q*)] with *D*_*q*_ and, thus, one learns almost nothing about the dynamics of the molecule of interest.

## V. CONCLUSIONS

Single-molecule force spectroscopy experiments are now able to probe the transitions of individual biomolecules over high activation barriers, making it possible to characterize transition paths and transition states in protein and nucleic acid folding.^6,7^ As we showed earlier for high barriers and soft linkers, the molecular transition rates can be estimated quite accurately over a relatively wide dynamic range,^1^ before slow apparatus response eventually dominates the apparent transition rate.

Transition paths are more sensitive to the apparatus dynamics in force spectroscopy experiments than transition rates. In the framework of a 2D model with anisotropic diffusion, we showed here that a mesoscopic pulling device attached to a rapidly relaxing molecule affects the observed transition paths even in the regime where the rates are accurate. In the limit of a slow apparatus coupled to a fast molecule, the 2D model can be reduced to a surface hopping model described by a set of coupled-reaction diffusion equations, Eqs. (12a) and (12b). This reduced model captures the physics of mesoscopic cantilevers (or beads) pulling molecular constructs via flexible polymeric linkers. The Brownian dynamics simulations showed that in this regime the full and reduced models are equivalent (as predicted by Berezhkovskii and Zitserman^20^) with the transition rates, distribution of transition path times, and mean transition path shape being the same within computational error.

Within the framework of a surface hopping model, we derived an analytic expression [Eq. (15)] for the transition path time. Importantly, this expression explicitly depends on the diffusion of the apparatus, *D*_*q*_, but is independent of the molecular diffusion coefficient, *D*_*x*_. By contrast, the molecular transition path time depends on *D*_*x*_, not on *D*_*q*_. In the limit of a slow apparatus, *D*_*x*_ ≥ *D*_*q*_, the predictions of the analytic expression agree well with the results from the simulations, with the mean transition path time, having an almost linear dependence on the size of the pulling device (or equivalently $D_{q}^{-1}$). In this regime, the diffusion constant extracted from the transition paths along the measured extension *q* is practically that of the apparatus (i.e., *D*_*q*_). A related problem is that, when the surface hopping model is valid, *q* trajectories contain little information about the rarely visited barrier region along the molecular extension *x* and one cannot reliably deconvolve the measured free energy profiles to obtain the molecular potential surface in the barrier region.^14^

The range of validity of the measured rates and transition path times depends on the properties of the apparatus, the linkers, and the molecule of interest, even when the observed trajectory of the total extension clearly indicates the presence of two or more well-defined states. The linkers should be soft in the sense that the ratio of the linker and molecular barrier stiffness is less than unity, a condition easily met. When the response of the apparatus is faster than that of the molecule, both the rates and transition path times are meaningful. When this is not the case, the measured transition paths do not accurately reflect the molecular ones. However, the rates can still be correct over a range of slow apparatus response when the molecular activation barrier in the presence of force is sufficiently large (greater than about 4 *k*_*B*_*T*).

Estimating the ratio *D*_*x*_/*D*_*q*_ is not entirely trivial. The experimentally accessible time scale of the fluctuations of the total extension *q* in the folded or unfolded states depends on both *D*_*q*_ and *D*_*x*_. The relaxation or correlation time of *q* in state *i* is defined by $\tau_{A}^{i}=\int_{0}^{\infty }{\left\langle \delta q\left(t\right)\delta q\left(0\right)\right\rangle }_{i}dt/{\left\langle \delta q^{2}\right\rangle }_{i}$, where *δq* = *q* − 〈*δq*〉_*i*_. For the 2D model, it can be shown that $\tau_{A}^{i}/{\left\langle \delta q^{2}\right\rangle }_{i}=1/D_{q}+1/\left(D_{x}{\left(1+G_{o}^{\prime \prime }\left(x_{i}\right)/\kappa_{l}\right)}^{2}\right)$. For soft linkers, when the hopping model is valid, $D_{q}\ll D_{x}{\left(1+G_{o}^{\prime \prime }\left(x_{i}\right)/\kappa_{l}\right)}^{2}$, and thus *D*_*q*_ can be determined^1^ as $D_{q}={\left\langle \delta q^{2}\right\rangle }_{i}/\tau_{A}^{i}$. In the opposite limit, if one can estimate *D*_*q*_ from the damped motion of the pulling apparatus without load (ignoring the effects of the linker), then *D*_*x*_ can be estimated using the above relaxation. Alternatively, if one is in the regime where molecular and observed rates are essentially equal and if one can obtain the free energy profile along *x* by deconvolution, then one can estimate^5^ *D*_*x*_ by equating the measured rate to the Kramers rate (assuming that the molecular extension is a good reaction coordinate). In any case, the direct experimental determination of *D*_*x*_ is difficult because *D*_*x*_ is an effective diffusion coefficient not given simply by the diffusion coefficient of the free polymer ends in the case of force-induced unfolding or rupture of compact molecular constructs. In simulations of a coarse-grained model of a small protein, the effective diffusion coefficient *D*_*x*_ for the end-to-end motion of the peptide chain was found to be slower by nearly a factor 100 in the folded state compared to that in the unfolded state.^22^ Using the Stokes-Einstein relation, a correction factor of 100 would bring the effective diffusion coefficient *D*_*x*_ for ∼1 nm sized amino acids into the range of the diffusion coefficient *D*_*q*_ of ∼0.1 *μ*m-sized beads. These simulations^22^ also suggest that the dynamics along *x* would be better described with a position-dependent diffusion coefficient, which is a further complication largely ignored here.

As a practical test to assess whether the measured quantities are suspect, one can use as a reference the apparatus-dominated 1D diffusion model along *q*. Both the diffusion coefficient *D*_*q*_ and the 1D free energy surface along *q* are experimentally accessible, the former from the *q*-fluctuations or damping coefficient and the latter from the *q* histogram (i.e., without deconvolution). One can then calculate the theoretical rates and transition path times for the 1D *q*-diffusion model. Agreement with the measured values indicates that the dynamic properties are strongly influenced by the apparatus and do not reflect those of the molecule.

We conclude that in typical force spectroscopy experiments the slow response of the apparatus affects the measured transition paths much more significantly than the transition rates. The distribution of transition path times, the mean transition path time, and the mean transition path shape strongly depend on the diffusion coefficient of the apparatus, even in a regime where the rates do not. Thus, extracting microscopic molecular properties from measured transition paths is challenging because the properties of the small molecule can be masked by the slow response of the pulling device.

## SUPPLEMENTARY MATERIAL

See supplementary material for the fit of the mean transition path shape for the 1D case allowing both *κ* and *D* to float and for an example of a time-averaged trajectory along the measured extension *q*.
